# High–Linear Energy Transfer Irradiation in Clinical Carbon-Ion Beam With the Linear Energy Transfer Painting Technique for Patients With Head and Neck Cancer

**DOI:** 10.1016/j.adro.2023.101317

**Published:** 2023-07-22

**Authors:** Ryosuke Kohno, Masashi Koto, Hiroaki Ikawa, Sung Hyun Lee, Kana Sato, Mitsuyasu Hashimoto, Taku Inaniwa, Toshiyuki Shirai

**Affiliations:** a Department of Accelerator and Medical Physics, Institute for Quantum Medical Science, National Institute for Quantum Science and Technology, Chiba, Japan; b Department of Radiologic Sciences, Graduate School of Health and Welfare Sciences, International University of Health and Welfare Graduate School, Tokyo, Japan; c QST Hospital, National Institute for Quantum Science and Technology, Chiba, Japan; d Department of Heavy Particle Medical Science, Graduate School of Medical Science, Yamagata University, Yamagata, Japan; e Department of Radiology, IUHW Narita Hospital, Chiba, Japan

## Abstract

**Purpose:**

Dose-averaged linear energy transfer (LET_d_) is one of the important factors in determining clinical outcomes for carbon-ion radiation therapy. Innovative LET painting (LP) has been developed as an advanced form of conventional intensity modulated carbon-ion radiation therapy (IMIT) at the QST Hospital. The study had 2 motivations: to increase the minimum LET_d_ (LET_dmin_) and to improve uniformity of the LET_d_ distribution within the gross tumor volume (GTV) by using LP treatment plans for patients with head and neck cancer while maintaining the relative biologic effectiveness (RBE)–weighted dose coverage within the planning tumor volume (PTV) the same as in the conventional IMIT plan.

**Methods and Materials:**

The LP treatment plans were designed with the in-house treatment planning system. For the plans, LET_d_ constraints and LET_dmin_, goal-LET_d_, and maximum-LET_d_ (LET_dmax)_ constraints for the GTV were added to the conventional dose constraints in the IMIT prescription. For 13 patients with head and neck cancer, the RBE-weighted dose to 90% (D90) and 50% (D50) of the PTV and the LET_dmin_, mean (LET_dmean_), and LET_dmax_ values within the GTV in the LP plans were evaluated by comparing them with those in the conventional IMIT plans.

**Results:**

The LP for 13 patients with head and neck cancer could keep D90s and D50s for the PTV within 1.0% of those by the conventional IMIT. Among the 13 patients, the mean LET_dmin_ of the LP plans for the GTV was 59.2 ± 7.9 keV/μm, whereas that of the IMIT plans was 45.9 ± 6.0 keV/μm. The LP increased the LET_dmin_ to 8 to 24 keV/μm for the GTV compared with IMIT.

**Conclusions:**

While maintaining the dose coverage to the PTV as comparable to that for IMIT, the LP increased the mean LET_dmin_ to 13.2 keV/μm for the GTV. For a GTV up to 170 cm^3^, LET_d_ > 44 keV/μm could be achieved using LP, which according to previous studies was associated with lower recurrence. In addition, the LP method delivered more uniform LET_d_ distributions compared with IMIT.

## Introduction

Carbon-ion radiation therapy (CIRT) has attracted significant interest because of its advantageous physical and radiobiological properties compared with photon-based therapy.^1^ Carbon ions exhibit a unique energy deposition in depth, known as the Bragg peak, where a small part of their energy is deposited at the beam entrance and most of the energy is released in the target when the ions are stopped. In addition, carbon ions possess a higher linear energy transfer (LET) than do photons and protons. The higher LET of carbon ions leads to a higher relative biologic effectiveness (RBE), causing clustered DNA damage 2 to 3 times more effectively than low LET radiations.^2^ Taking advantage of these physical and radiobiological properties, CIRT has provided promising treatment by delivering higher RBE-weighted doses to targets while reducing the doses to organs at risk (OARs).^3^^,^^4^

The relationship between the dose-averaged LET (LET_d_)^5^ distribution within a tumor and tumor control has been explored in CIRT in several recent studies. Hagiwara et al^6^ reported for pancreatic cancer that there was a significant association with local control when the LET_d_ in the gross tumor volume (GTV) was greater than 44 keV/μm. Matsumoto et al^7^ also reported that the LET_d_ distribution in a tumor was associated with local control in chondrosarcomas, and patients treated with ion beams of higher LET_d_ might have an improved local control rate for unresectable chondrosarcomas. The LET_d_ is one of the important factors to improve local control for cancer. In contrast, regarding normal tissue, the RBE-weighted dose for 132 uterine carcinomas had a greater influence on rectal complication than LET_d_.^8^

For head and neck cancers, the therapeutic effectiveness and safety of CIRT has been reported.^9^, ^10^, ^11^ Koto et al^11^ reported that the 2-year overall survival and local control rates of patients with sinonasal cancer were 79.6% and 84.1%, respectively. Regarding acute toxic effects, they observed grade 3 mucositis in 19% of the patients and grade 3 dermatitis in 3%. In addition, 17% of the patients developed grade 3 and 4 late toxic effects, of which visual impairment was the most common.

To further enhance local tumor control, dose escalation may be the most common strategy. However, due to the occurrence of adverse effects, it is difficult to put further dose escalation into practice for head and neck cancers in CIRT. Other irradiation techniques are thus required for improved local tumor control. Bassler et al^12^ have proposed an innovative LET painting (LP) method that redistributes the LET of carbon-ion beams. The LP can maximize the LET in a specific region of the target and is thus useful to overcome tumor hypoxia. The LP should also be useful to improve the local tumor control for head and neck cancers.

In this study, the authors investigated Bassler et al's^12^ proposal as a new irradiation technique aimed at maximizing the therapeutic potential of CIRT for head and neck cancers. An in silico LP planning study was carried out, and it was verified that the LP could achieve higher LET_d_ for a target in clinical cases while maintaining dose coverage to the planning tumor volume (PTV) and dose deposition to the OARs. Finally, RBE-weighted dose and LET_d_ distributions with the LP for patients with head and neck cancer were compared with those with the conventional intensity modulated carbon ion radiation therapy (IMIT).

## Methods and Materials

### Patient cases

A total of 13 patients with head and neck cancers who received CIRT at the QST Hospital were investigated. Their tumor sites were the maxillary sinus, nasal cavity, ethmoid sinus, sphenoid bone sinus, skull base, parotid gland, and middle ear. The size of the GTV and PTV ranged from 5.9 to 143.1 cm^3^ and from 29.41 to 287.97 cm^3^, respectively. The prescription of RBE-weighted doses ranged from 60.8 to 70.4 Gy (RBE) at 3.8 to 4.4 Gy (RBE) per daily fraction.

### Treatment planning system

Treatment plans were designed with the treatment planning software XiDose. The XiDose was developed at the QST as part of our collaboration with Elekta AB, Sweden, for scanned carbon-ion radiation therapy.^13^ The analytical pencil-beam algorithm with the trichrome beam model^14^ in XiDose was adopted for patient dose calculations. In the beam model, different LET_d_ values were assigned to the 3 Gaussian components to represent the radial variation of the LET_d_ over the beam cross section. A new function of the LP was integrated into XiDose.

The dose deposited by the scanned pencil beam at the *j*th spot to location *r, D_j_*(*r*), in water can be obtained from the Monte Carlo simulation software PTSsim^15^ as

$$D_{j}\left(r\right)=\sum_{k}E_{jk}\left(r\right),$$

where *Ejk*(*r*) is the energy imparted to *r* in the *k*th energy-deposition event by the *j*th beamlet. All primary and secondary particles were included in this calculation. All ions were tracked down to the 0 range, whereas secondary electrons were forced not to be generated to shorten the computation time. Similarly, the LET_d_ of the *j*th beamlet, *LETd_j_*(*r*), in water was calculated as

$$LETd_{j}\left(r\right)=\frac{\sum_{k}E_{jk}\left(r\right)\cdot S_{jk}\left(r\right)}{\sum_{k}E_{jk}\left(r\right)}$$

where *s_jk_*(*r*) is the stopping power of water for the ion contributing to the *k*th energy-deposition event, calculated with the stopping power calculation program MSTAR.^16^ The biologic dose distribution by the modified microdosimetric kinetic model^17^ can be optimized through inverse treatment planning.

The IMIT prescription is a group of interrelated instructions that define the limits, priorities, and goals for the optimization algorithm. The IMIT prescriptions of XiDose include several different kinds of dose- and LET_d_-objectives, desired goals, and their relationships for both targets and OARs. XiDose can optimize the RBE-weighted dose and LET_d_ distributions in a patient simultaneously, using IMIT or LP with carbon ions.

### LET painting treatment planning

First, the conventional IMIT treatment plan was designed as usual, because the LP treatment plan was based on the IMIT plan. The beam angle, the number of beams, and the dose constraints for the GTV, PTV, and OARs were identical to those of actual CIRT treatments. The IMIT plans consisted of 2 to 4 beams in this study.

Next, the LP treatment plan was designed. In this study, because the goal was to evaluate purely the LP plans in the same beam conditions as the conventional IMIT plans, the same beam arrangement and the dose constraints as the corresponding IMIT plan were set to the LP plan. To optimize LET_d_ within the GTV, only the LET constraints were added to the LP prescription, that is, the minimum- (LET_dmin_), the goal-, and the maximum-LET_d_ (LET_dmax_) constraints. For the LET_dmin_ , goal-, and LET_dmax_ constraints, *L-5, L*, and L+10 keV/μm were set, respectively. The total optimization time for the LP took about 1.5 times longer than that for the IMIT.

Although an input of 100 keV/μm instead of *L*+10 was preliminarily tested for the LET_dmax_ constraint, the LET_dmin_ within the GTV was not increased significantly owing to the LET_dmin_ constraints: *L-5.* On the other hand, LET_dmax_ values increased over 100 keV/μm in an effort to satisfy the 100 keV/μm of the LET_dmax_ constraint. As a result, the uniformity of the LET_d_ distribution deteriorated. Therefore, the LET_dmax_ constraint of *L*+10 was adopted in the following LP treatment planning studies.

To investigate how much the LET_dmin_ to the GTV can be increased while maintaining the dose coverage to the PTV compared with the corresponding IMIT plan and delivering clinically acceptable OAR doses, the *L* was varied from 45 to 105 keV/μm at a 10-keV/μm interval in the LP plans for the 13 patients with head and neck cancer. Basically, calculation of the LP optimizations would be stopped if the RBE-weighted dose to 90% of the PTV (PTV D90) of the LP plans deteriorated over 5% compared with the IMIT plans. To assist in observing the deterioration tendency of PTV D90, results associated with considerably low PTV D90 values for patients 8 and 10 were also included.

### RBE-weighted dose and LET_d_ evaluation for LP treatment planning

To evaluate usefulness of the LP plans, their RBE-weighted dose and LET_d_ distributions were investigated for the 13 patients. The LP plans with a small reduction within 1.0% in the RBE-weighted dose to the PTV D90 compared with the IMIT plans were used in this study. The PTV D90, the RBE-weighted dose to 50% of the PTV (PTV D50), and the maximum dose to the PTV in the LP plans were determined and compared with those in the IMIT plans.

Then, the LET_dmin_, the mean LET_d_ (LET_dmean_), and the LET_dmax_ for the GTV in the LP plans were determined and compared with those in IMIT. The GTV size dependence on the LET_dmin_, LET_dmean_, and LET_dmax_ for each patient was also investigated. Lastly, the maximum dose and the LET_dmax_ for the OARs, stem, chiasma, and optic nerves for the healthy side were evaluated by comparing them with those in the IMIT plans.

## Results

**Figure 1:**
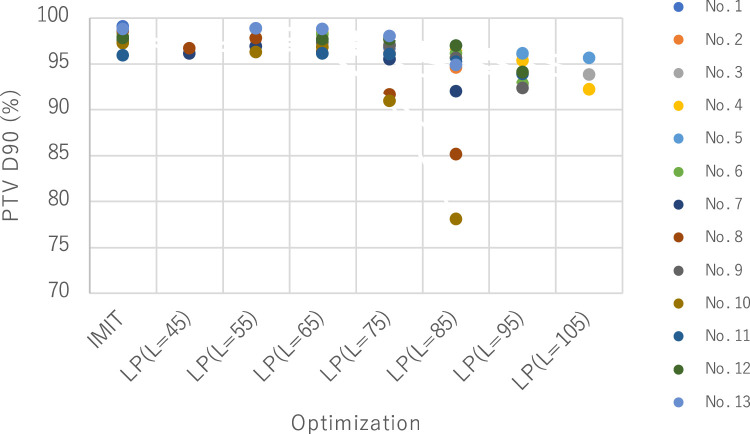
PTV D90 of IMIT and LP (*L* = 45, 55, 65, 75, 85, 95, and 105) for patients 1 to 13; 100% was defined as the prescribed dose Gy (RBE). *Abbreviations:* D90 = percentage of dose to 90%; IMIT = intensity modulated carbon-ion radiation therapy; LP = linear energy transfer painting; PTV = planning tumor volume; RBE = relative biologic effectiveness.

Figure 1 shows the PTV D90 of IMIT and LP (*L* = 45, 55, 65, 75, 85, 95, and 105 keV/μm). The prescribed RBE-weighted dose Gy (RBE) was set as 100%. The PTV D90 deteriorated with the increase of the goal-LET_d_ constraint *L*, especially when it was greater than 75 keV/μm. This was why the dose coverage for the PTV was deteriorated to satisfy the LET_dmin_ and goal-LET_d_ constraints. This result indicated the difficulty in delivering the LET_dmin_ of greater than 70 keV/μm to the GTV while maintaining the dose coverage to the PTV in the LP plans of head and neck cancers.

**Figure 2:**
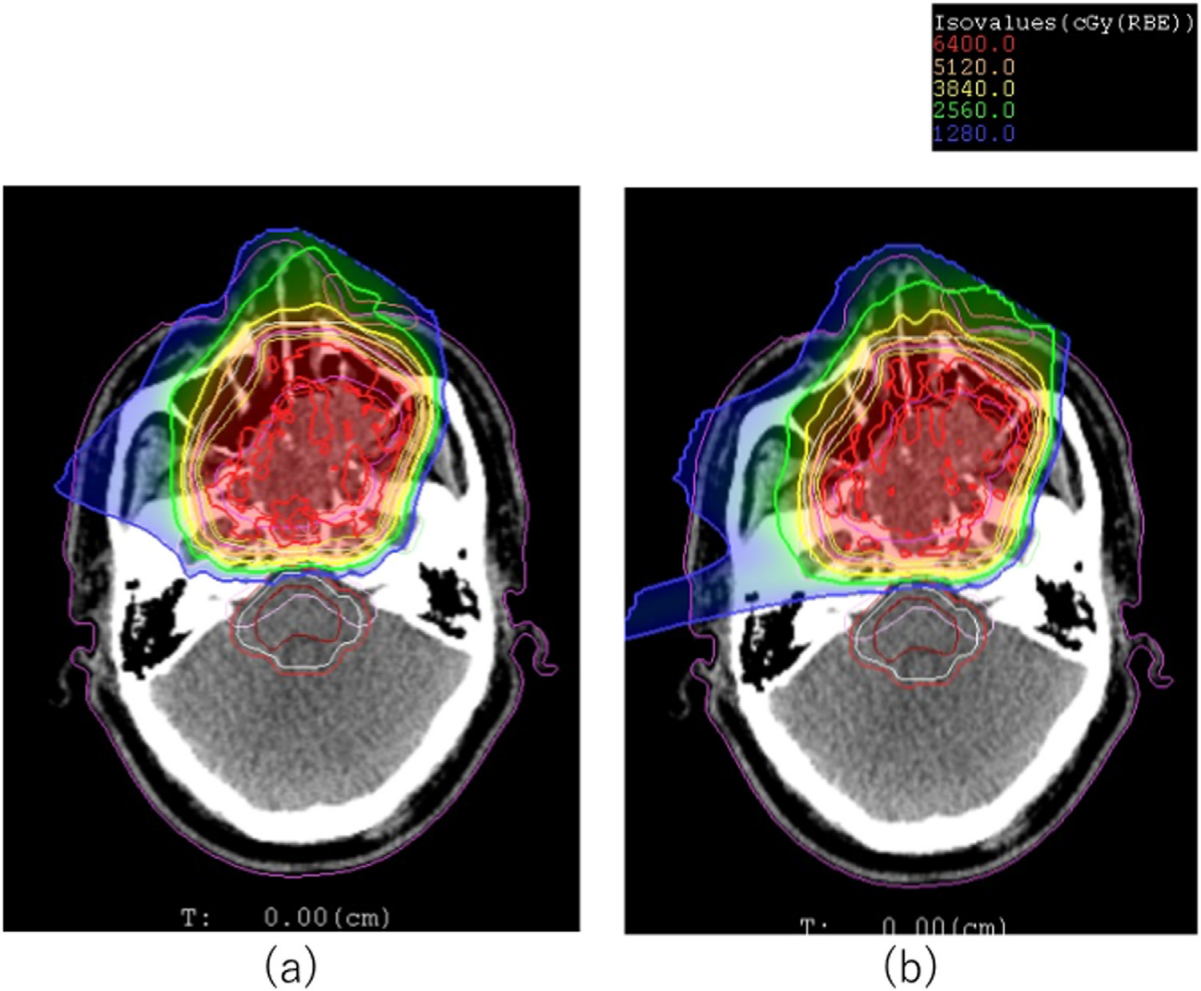
Relative biologic effectiveness–weighted dose distributions of (a) intensity modulated carbon-ion radiation therapy and (b) linear energy transfer painting.

**Figure 3:**
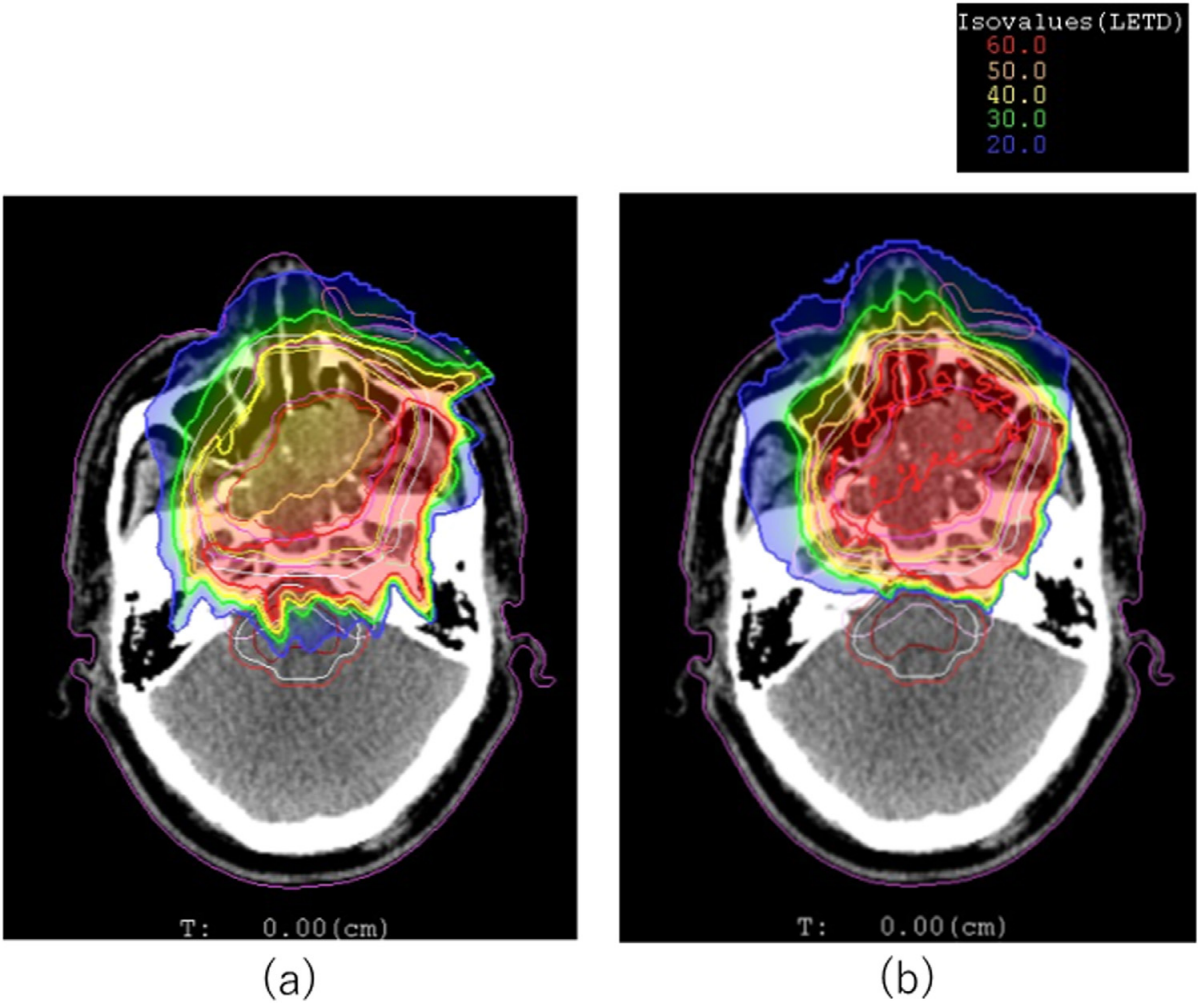
Dose-averaged linear energy transfer distribution of (a) intensity modulated carbon-ion radiation therapy and (b) linear energy transfer painting.

Figures 2 and 3 are comparisons of typical RBE-weighted dose and LET_d_ distributions for (2a and 3a) IMIT and (2b and 3b) LP for patient 10, whose GTV was 71.31 cm^3^. Four beams of 15°, 255°, 285°, and 345° were used for the IMIT and LP plan, and the LP plan was optimized with *L* = 65 keV/μm. For patient 10, the PTV D90, PTV D50, and maximum dose in IMIT were 97.3%, 99.9% and 108.1%, respectively. Corresponding doses in the LP were 96.9%, 99.4% and 104.4%. No significant difference in dose coverage to the PTV was observed among them.

A higher dose was observed around the right ear in Fig. 2b for the LP, although it was less than the tolerance dose to the ear of 40 Gy (RBE). This was because the fact that the beam weight for the 255° beam in the LP plan was higher than that of the IMIT plan to increase the LET_d_ in the GTV. Thus, although no critical increase in dose for OARs was seen in this case, the LP in some clinical cases might have an unfavorable cost, which is an increased dose in the entry channels. For the 13 patients with head and neck cancer, the mean difference in PTV D90 and PTV D50 between the LP and IMIT was –0.39 ± 0.3% and –0.11 ± 0.2%, respectively. This meant that the LP delivered clinically comparable dose distributions to the target the same as IMIT did.

The LET_dmin_, LET_dmean_, and LET_dmax_ within the GTV in IMIT for patient 10 were 45 keV/μm, 55 keV/μm, and 117 keV/μm, respectively. On the other hand, in the LP, their respective values were 58 keV/μm, 62 keV/μm, and 90 keV/μm. It was obvious that the LP delivered high and uniform LET_d_ values throughout the GTV, whereas high LET_d_ values were delivered only to the posterior part of the tumor in IMIT.

**Figure 4:**
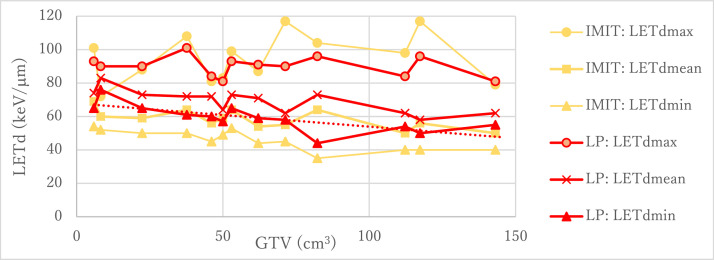
LET_dmin_, LET_dmean_, and LET_dmax_ of the IMIT and LP versus GTV size (Volume) for 13 patients with head and neck cancer. The dotted line is a linear fitting function obtained from the LET_dmin_ results of the LP and given as LET_dmin_ keV/μm = –0.139 keV/μm/cm^3^ × *V* + 67.8 keV/μm. *Abbreviations:* GTV = gross tumor volume; IMIT = intensity modulated carbon-ion radiation therapy; LET = linear energy transfer; LET_dmax_ = maximum dose-averaged LET; LET_dmean_ = mean dose-averaged LET; LET_dmin_ = minimum dose-averaged LET; LP = LET painting.

Figure 4 shows the LET_dmin_, LET_dmean_, and LET_dmax_ of the LP and IMIT as a function of GTV size for 13 patients with head and neck cancer. The LET_d_ values in the LP and IMIT ranged from 44 to 101 keV/μm and from 35 to 117 keV/μm, respectively. The mean LET_dmin_ values for the GTV in the respective LP and IMIT plans were 59.2 ± 7.9 and 45.9 ± 6.0 keV/μm. The LP increased the LET_dmin_ to 8 to 24 keV/μm (average, 13 keV/μm) for the GTV compared with IMIT. The mean LET_dmean_ values and LET_dmax_ values in both plans were 69.2 ± 7.0 and 58.5 ± 5.7, and 90.0 ± 6.1 and 94.9 ± 14.5 keV/μm.

The LP could increase LET_dmin_ and decrease LET_dmax_, compared with IMIT. The mean LET_d_ differences between the LET_dmax_ and LET_dmin_ in the LP and IMIT were 30.8 ± 10.1 and 49.0 ± 16.8 keV/μm. When looking at the LET_dmin_ for the LP, it was observed that the LET_dmin_ tended to decrease with the increase of the GTV size with a linear regression line for LET_dmin_ keV/μm = –0.139 keV/μm/cm^3^ × *V* + 67.8 keV/μm, where *V* is the GTV size. The LET_dmin_ could be estimated roughly from the GTV size by this approximate expression.

**Figure 5:**
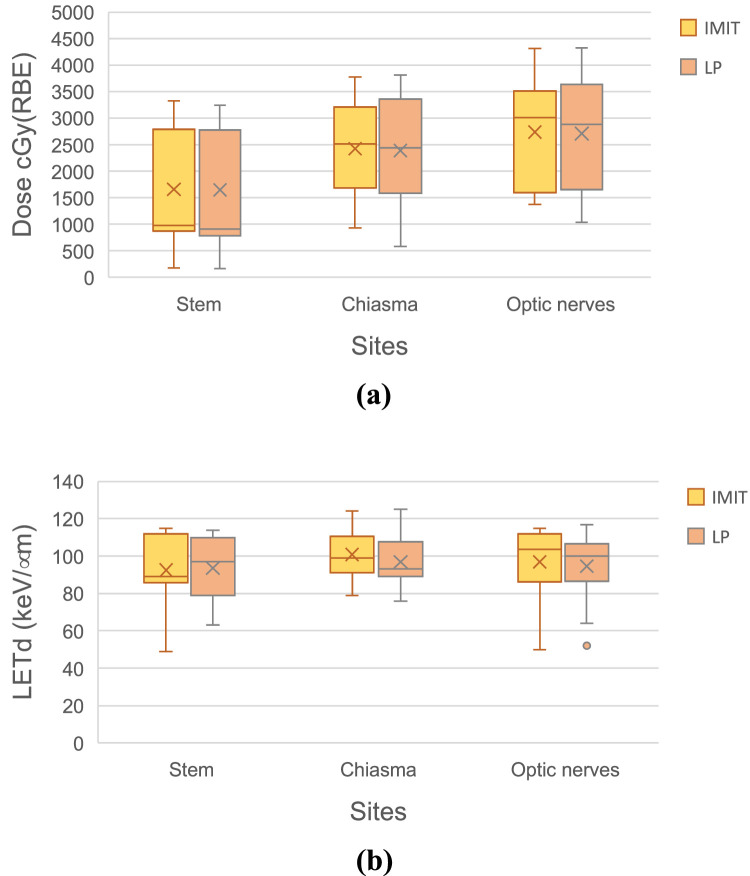
Box plots of (a) the maximum RBE-weighted dose and (b) the LET_dmax_ to stem, chiasma, and optic nerves for the IMIT and LP for 13 patients with head and neck cancer.

Figure 5 gives box plots of the (5a) maximum RBE-weighted dose and (5b) LET_dmax_ to OARs, the stem, the chiasma, and the optic nerves for IMIT and LP for the 13 patients with head and neck cancer. No differences in the maximum RBE-weighted doses and LET_dmax_ values for OARs were seen between IMIT and LP.

## Discussion

For 13 patients with head and neck cancer, the LP could achieve PTV D90 and PTV D50 within differences of less than 1.0% compared with those of IMIT, and the LP could increase the mean LET_dmin_ to 13.2 keV/μm for the GTV. It was obvious that the LP could safely increase the LET_dmin_ in the GTV while maintaining the dose coverage for the PTV. The latest clinical analysis for patients with head and neck cancer reported that the LET_d_ was one of the important factors for tumor control, although the RBE-weighted dose contributed to normal-tissue complication.^18^ Hence, the LP, which can give the higher LET_d_ to the target, may improve tumor control.

As shown in Fig. 1, the PTV D90 for the LP plan deteriorated with the increase of the goal-LET constraint *L* in the LP prescription. *L* could be set to 75 keV/μm for most patients with head and neck cancer. Consequently, the LET_dmin_ for the GTV in the LP plan could be increased to approximately 60 keV/μm. This LET_d_ value is sufficiently high compared with the threshold LET_d_ values of recurrence (ie, 44 keV/μm for pancreatic cancers and 40 keV/μm for unresectable chondrosarcomas) reported in previous clinical studies.^5^^,^^6^ However, more importantly, it is necessary to know how large the LET_dmin_ value must be to effectively control the head and neck tumors. Certainly, Kohno et al^18^ found that recurrence was observed in patients with head and neck adenoid cystic carcinoma when the LET_dmin_ in the GTV was lower than 42 keV/μm. Further retrospective analyses of patients with head and neck cancer treated by CIRT in terms of the LET_d_ are ongoing.

The LET_dmin_ tends to decrease with the increase of GTV size, as shown in Fig. 4. For the GTV of 200 cm^3^, the LET_dmin_ realized by the LP plan was estimated to be 40 keV/μm. For such large tumors, even the LP with carbon ions may be insufficient to deliver an LET_dmin_ exceeding the threshold LET_d_ of recurrence. Therefore, studies on multi-ion technique with heavier ions (eg, oxygen or neon ions) have been in the spotlight also.^13^^,^^19^, ^20^, ^21^ An intensity modulated composite particle therapy using helium and carbon, oxygen, and neon ions for prostate cancer could improve the PTV LET_dmin_ to 80 keV/μm while maintaining the dose in the PTV to a uniform 2 Gy.^21^ Therefore, multi-ion radiation therapy may allow for even greater treatment potential in radioresistant cancers.

## Conclusion

The LP could increase the LET_dmin_ of the GTV up to 13.2 keV/μm compared with conventional IMIT while maintaining the PTV D90 and PTV D50 with differences within 1.0% compared with those with IMIT. For a GTV up to 170 cm^3^, LET_d_ > 44 keV/μm could be achieved using LP, which, according to previous studies, was associated with lower recurrence. For the OARs, no differences for the maximum RBE-weighted doses and LET_dmax_ values could be seen between the IMIT and LP plans. The authors are planning to evaluate the safety and usefulness of the LP with a clinical trial in the near future.

## Disclosures

The authors declare that they have no known competing financial interests or personal relationships that could have appeared to influence the work reported in this paper.
